# Microfluidic channel optimization to improve hydrodynamic dissociation of cell aggregates and tissue

**DOI:** 10.1038/s41598-018-20931-y

**Published:** 2018-02-09

**Authors:** Xiaolong Qiu, Jen-Huang Huang, Trisha M. Westerhof, Jeremy A. Lombardo, Katrina M. Henrikson, Marissa Pennell, Pedram P. Pourfard, Edward L. Nelson, Pulak Nath, Jered B. Haun

**Affiliations:** 1 Department of Biomedical Engineering, Henry Samueli School of Engineering, University of California Irvine, Irvine, CA 9269 USA; 2 Department of Chemical Engineering and Materials Science, Henry Samueli School of Engineering, University of California Irvine, Irvine, CA 92697 USA; 3 Department of Medicine, Division of Hematology/Oncology, School of Medicine, University of California, Irvine, Irvine, CA 92697 USA; 4 Department of Molecular Biology and Biochemistry, Ayala School of Biological Sciences, University of California, Irvine, Irvine, CA 92697 USA; 5 Chao Family Comprehensive Cancer Center, University of California Irvine, Irvine, CA 92697 USA; 6 Department of Chemical Engineering, National Tsing Hua University, Hsinchu, Taiwan; 7 Applied Modern Physics, Los Alamos National Laboratory, Los Alamos, NM 87545 USA

## Abstract

Maximizing the speed and efficiency at which single cells can be liberated from tissues would dramatically advance cell-based diagnostics and therapies. Conventional methods involve numerous manual processing steps and long enzymatic digestion times, yet are still inefficient. In previous work, we developed a microfluidic device with a network of branching channels to improve the dissociation of cell aggregates into single cells. However, this device was not tested on tissue specimens, and further development was limited by high cost and low feature resolution. In this work, we utilized a single layer, laser micro-machined polyimide film as a rapid prototyping tool to optimize the design of our microfluidic channels to maximize dissociation efficiency. This resulted in a new design with smaller dimensions and a shark fin geometry, which increased recovery of single cells from cancer cell aggregates. We then tested device performance on mouse kidney tissue, and found that optimal results were obtained using two microfluidic devices in series, the larger original design followed by the new shark fin design as a final polishing step. We envision our microfluidic dissociation devices being used in research and clinical settings to generate single cells from various tissue specimens for diagnostic and therapeutic applications.

## Introduction

Recent insights into the importance of cellular heterogeneity and rare driver cells have combined with advancements in sequencing and molecular detection technologies to help usher in the era of single cell diagnostics^1–5^. This has led to ambitious efforts such as the Human Cell Atlas initiative to identify and characterize cell types within the body^6,7^, as well as potential use of single cell functional information for diagnosing and treating diseases^5,8–13^. However, most cells reside within tissue masses and organs, and thus significant effort in front-end tissue dissociation is required prior to single cell analysis^2^. Current tissue dissociation procedures involve mincing tissues into small pieces with a scalpel, lengthy digestion with proteolytic enzymes, and mechanical treatment by pipetting and/or vortexing. This is a laborious, time-consuming, and inefficient process that often results in incomplete extraction of all single cells from a given tissue sample. Specifically, long digestion times can lead to poor cell quality vis-a-vis changes in molecular expression profiles and/or death. Thus, improving tissue dissociation such that single cells can be liberated in a rapid, gentle, and thorough manner would dramatically advance the clinical potential of single cell diagnostics under modalities such as flow cytometry, mass spectroscopy, and single cell sequencing^1,2,14,15^. The fields of tissue engineering and regenerative medicine would also directly benefit from improving the procurement of healthy and functional primary, progenitor, and stem cells from various organs and tissues to serve in tissue constructs and cell-based therapies^16–22^.

Microfluidic device technologies have been developed to aid dissociation, with early works focused on digesting, cutting, or physically breaking down tissues or cellular aggregates^23–25^. However, these devices either were not specifically designed to produce single cells, or suffered from significant clogging issues. In previous work, we developed a novel microfluidic device to gradually break down cellular aggregates all the way down into single cells in a rapid and efficient manner^26^. Key features included an array of branching channels that decreased in size from millimeters to hundreds of microns, as well as repeating expansions and constrictions of the channel width that generated hydrodynamic fluid jets. The net effect was that shear stresses of different size scales and magnitudes were applied to cell aggregates and clusters to mechanically separate cells from each other. Extensive testing with cancer cell aggregates and spheroids demonstrated that our microfluidic device significantly improved cell recovery in terms of single cell numbers and purity. These results were obtained using minimal proteolytic digestion, and in some cases even without the use of enzymes. Moreover, we did not observe changes in cell viability, and total processing time was less than 10 minutes. However, to date we had not tested this device on actual tissue specimens, which would still require off-chip mincing and digestion prior to mechanical dissociation. Furthermore, we fabricated our devices from multiple layers of hard plastic using a commercial laminate process. While this provided a robust device that was amenable to large-scale manufacturing, further device development was limited by high fabrication cost and the poor resolution of commercial lasers. Thus, a rapid prototyping method is needed to optimize microfluidic channel design and improve dissociation performance.

Rapid prototyping of microfluidic devices has been dominated by photolithography and molding of polydimethylsiloxane (PDMS) because it is fast, inexpensive, easy to use, and has relatively high resolution. However, the elastic nature of PDMS is a drawback for applications that require high fluid flows or pressures^27^, such as tissue dissociation. Moreover, fabrication methods that use template molding are not ideal for multilayer devices or large scale manufacturing. Laser micro-machined polymeric films in conjunction with adhesive transfer tapes for bonding have recently emerged as a robust and cost-effective rapid prototyping method for microfluidic devices^28^. The versatility and efficacy of such devices has been demonstrated in an array of microfluidic applications, with channel resolutions on the order of 50 µm^29–35^. Most importantly, the use of laser micro-machined polymeric films and adhesive transfer tapes will recapitulate the form and function of the commercial laminate systems used for our original microfluidic dissociation device.

In this work, we utilize laser micro-machined polyimide films as a rapid prototyping tool to optimize the design of our microfluidic cell aggregate dissociation device. We first demonstrate that a single-layer polyimide film based device can dissociate cancer cell aggregates into single cells, but not as effectively as a multilayer counterpart. However, reducing channel dimensions improves performance such that dissociation efficiency is on par with, and even surpasses, our multilayer device. Next we employ computational fluid dynamics simulations to optimize the design of the channel expansions, resulting in a new geometry that we refer to as a shark fin. While the shark fin design modestly improves cancer cell aggregate dissociation, extending the extent of the shark fin channel expansions yields the best dissociation results that we have seen to date. Finally, we validate performance of our microfluidic dissociation devices using mouse kidney tissue. Following standard tissue mincing and collagenase digestion procedures, we find that microfluidic treatment dramatically increases single cell recovery, lowers the number of aggregates, and maintains cell viability. The best results are obtained using two microfluidic devices in series, with the original multilayer device first reducing large tissue fragments and aggregates into smaller units, followed by a final sample polishing step using the new optimized single layer shark fin device to produce more single cells. Compared to the standard method of enzymatically digesting for 1 hour, the dual device treatment produces three-fold more single cells at the same digestion time, and comparable numbers after only a brief 15 min digestion. This work establishes, for the first time, that microfluidic dissociation improves the speed and efficiency of single cell production from digested tissue. Moreover, the combination of different devices and operating principles is needed when working with tissue so as to span a large enough range of channel sizes and shear forces. In future studies, we will continue to explore new dissociation device designs, as well as integration with other microfluidic operations to enable further cell processing and analysis. We will also examine performance using different tissue types including various normal organs and solid tumor specimens.

## Results and Discussion

### Device design

**Figure 1:**
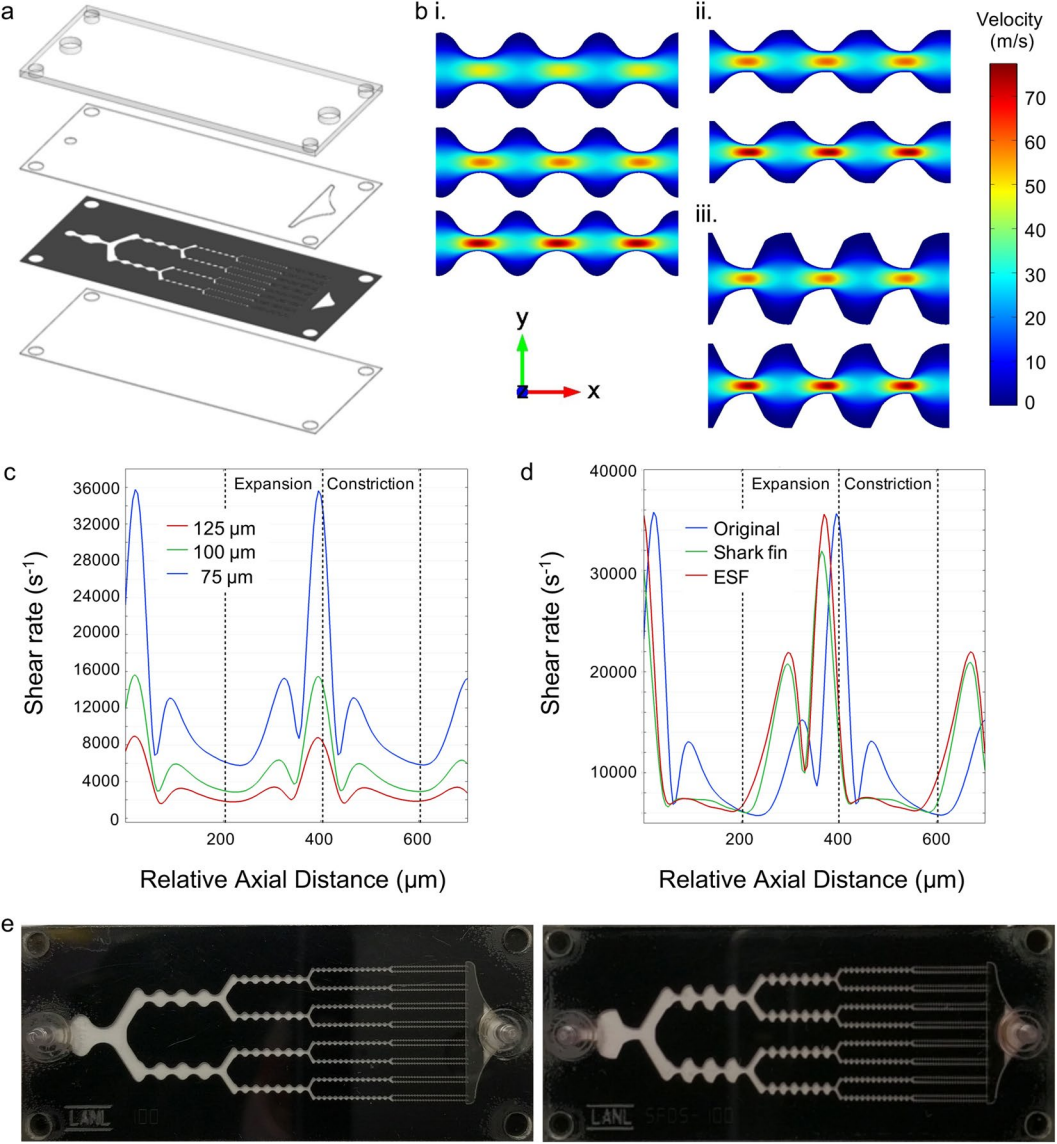
Single layer, polyimide based microfluidic device with new channel designs. (**a**) Exploded view showing the four device layers including, from top to bottom, acrylic top layer, PET outlet layer, channel layer produced by laser-etching polyimide film, and PET bottom layer. Adhesive transfer tapes were laminated to the PET layers and used to hold the device together. Holes used to attach hose barbs are shown in the top layer. (**b**) Finite-element fluid dynamics simulations showing velocity profiles for the (i) original, (ii) shark fin, and (iii) extended shark fin designs at (top to bottom) 125, 100, and 75 μm minimum widths. Simulations were performed at 1 mL/min. Decreasing channel width led to higher maximum flow velocities in the channel constrictions, while the extended shark fin provided greater variation in flow rate between the expansions and constrictions. (**c**) Shear rate at the device centerline for the original design at different channel widths. Three shear rate peaks can be seen, all of which increase as width becomes smaller. The major peak corresponds to fluid acceleration between the expansions and constrictions, and the two smaller peaks are due to fluid deceleration within the separate regions. (**d**) The shark fin and ESF designs enhance shear rate due to fluid deceleration within expansions, but there is no longer a second peak within constrictions. (**e**) Pictures of fabricated single layer devices with (left) original and (right) extended shark fin designs. Both devices had a minimum channel width of 100 µm in the fifth stage.

Our original microfluidic dissociation device design consisted of a network of branching channels that progressively decreased in channel width, and channels were arranged across three layers of hard PET plastic that were separated by via layers^26^. The channels also contained repeated expansions and constrictions that generated fluidic jets to supply the shear force needed to separate cells from each other. In this work, we employed laser micro-machined polyimide films and adhesive transfer tapes as a rapid prototyping method to optimize both channel width and the shape of the channel expansions to improve dissociation performance. Devices were fabricated using four layers of polymeric films/sheets that were each bonded by adhesive transfer tape (Fig. 1a). Microfluidic channel features were laser micro-machined into polyimide films, which provided far superior feature resolution than was achieved in the multilayer device (see Supplementary Information, Fig. S1). We also explored new channel designs, such as reducing the minimum channel width from the original value of 125 µm in the final stage down to 100 and 75 µm. Channel dimensions within stage 5 are shown for all three designs in the Supplementary Information, Fig. S2). Note that all channel scaling ratios were retained from the original device design, including minimum channel width between different branch stages (factor of 2) and the expansion to constriction width within each stage (factor of 3). Based on computational fluid dynamics (CFD) simulations, decreasing channel width to 100 and 75 µm would increase maximum flow velocity within the channel constrictions by 15 and 65%, respectively (Fig. 1bi). Since fluid shear scales with both the increasing velocity and decreasing channel dimensions, it would increase by 50 and 150%, respectively. We also calculated the shear rate at the centerline arising from flow gradients in the axial and cross-sectional dimensions, and results are displayed in Fig. 1c. Decreasing channel width to 100 and 75 µm increased maximum shear rate by 90 and 350%, respectively. Interestingly, shear rate was highest in the transition from expansion to constriction, as the fluid was accelerated to its maximal value. Two additional regions of moderate shear rate were also present, which corresponded to separate stages of fluid deceleration within the expansion and constriction regions. Based on these observations, we hypothesized that cell aggregate dissociation could be enhanced by combining the two smaller shear rate peaks from deceleration into a single, larger peak. Therefore, we explored new expansion region designs using CFD simulations and identified a non-symmetric geometry characterized by a more gradual initial expansion followed by a more abrupt constriction that we called a shark fin (Fig. 1bii). The shark fin design did not alter maximum flow velocity or shear rate values, as these were dictated by the minimum channel width found at the constriction. However, the shark fin increased the magnitude of the shear rate peak within the expansion region by 50% (Fig. 1d). It should be noted that the second deceleration peak in the constriction region was lost. Finally, we also created an extended shark fin (ESF) design by doubling the slope of the shark fin design constriction (Fig. 1biii), which further increased shear rate upon entering the expansion region (Fig. 1d). In summary, the shark fin and ESF designs provided two regions of high shear rate, in comparison to one large and two moderate shear rate regions for the original device. Channel dimensions within stage 5 are shown for the shark fin and ESF designs in Supplementary Information, Fig. S2. Pictures of the single layer polyimide dissociation devices with the original and extended shark fin channel designs are shown in Fig. 1e.

### Evaluation of single channel layer devices with different channel widths

**Figure 2:**
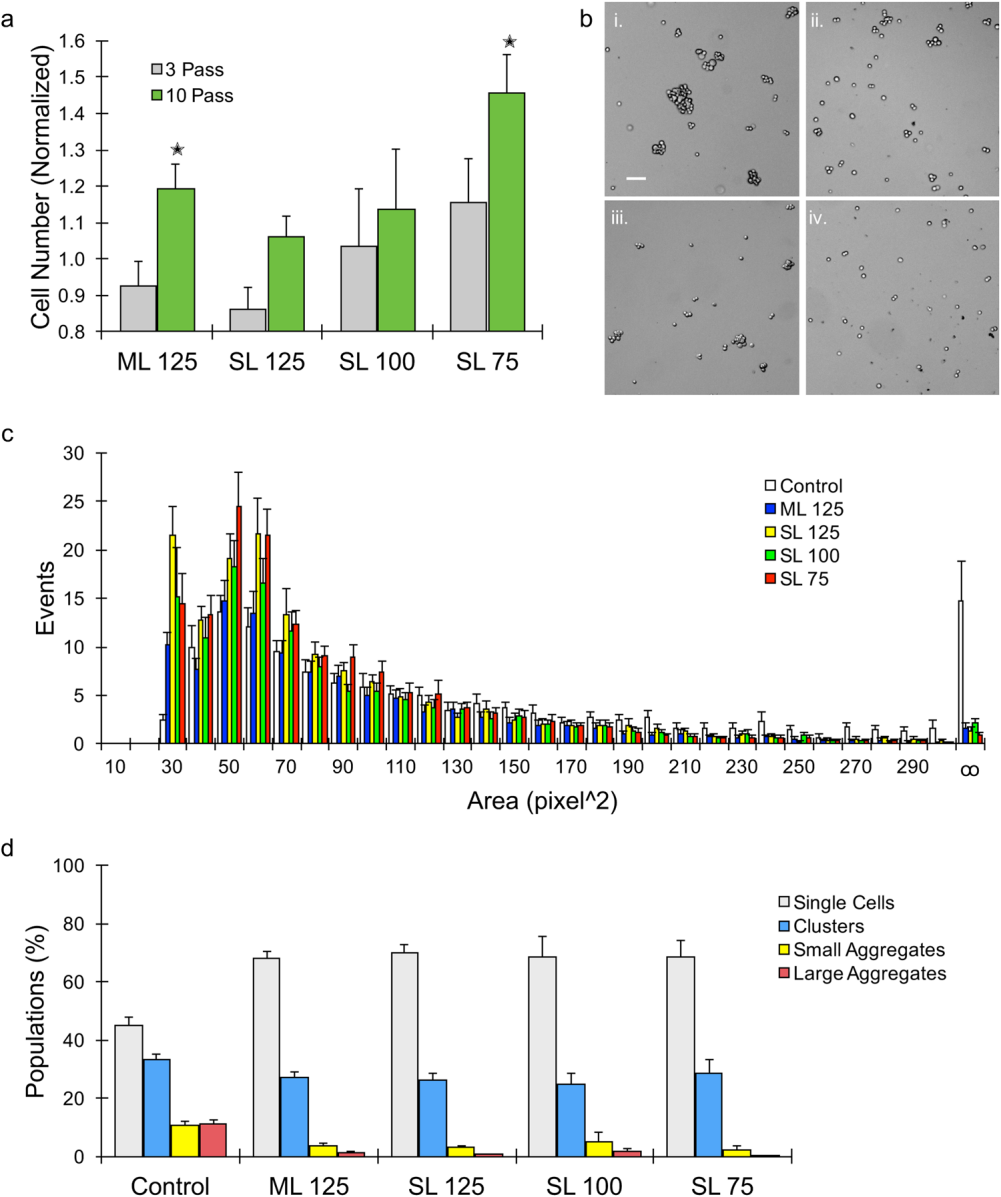
Evaluation of single layer devices at different channel widths. (**a**) MCF7 breast cancer cells recovered in device effluents were quantified and normalized by the initial value prior to device processing. The original multilayer (ML) device significantly improved single cell yield after 10 passes, but this was not seen in the single layer (SL) device with the same channel dimensions (125 µm). However, decreasing channel width improved performance, with the SL device with 75 µm width exceeding the ML case. **(b**) Micrographs showing cell suspensions (i) prior to device processing and (ii-iv) after passing 10 times through the (ii) ML, (iii) SL 125, and (iv) SL 75 devices. Scale bar is 100 μm. (**c**) Cell unit area histogram determined by analyzing micrographs, leading to classification of single cells (<80 pixels^2^ and aspect ratio <1.2), clusters (80 to 200 pixels^2^ and aspect ratio >1.2; 2–3 cells), small aggregates (200 to 300 pixels^2^, 4–10 cells), and large aggregates (>300 pixels^2^, >10 cells). Data was combined between 3 and 10 pass conditions. (**d**) Cell populations above plotted as percent total for the control, ML device, and various SL devices after 10 passes. Device processing eliminated almost all large aggregates and most small aggregates, leading to higher single cell purity. Clusters only decreased slightly. Error bars represent standard errors from at least three independent experiments. *indicates p < 0.05 relative to non-device processed control (value = 1).

We first compared the performance of the original channel design in the new single layer device to the original multilayer version. We used MCF7 human breast cancer cell line monolayers for these studies, which are strongly cohesive and produce large numbers of aggregates after routine cell culture and trypsinization. As in previous work, cells were suspended in buffer and introduced into the microfluidic devices using a syringe pump at 12.5 mL/min, and were passed through the device either 3 or 10 times. Device effluents were recovered and cell numbers were quantified using a cell counter. For the multilayer device, no change in cell recovery was observed after 3 passes, but 10 passes generated 20% more cells (Fig. 2a). The single layer device did not show a significant change in cell number for either case. Thus, simplifying the design to a single channel layer substantially diminished dissociation power. This was most likely due to the fact that samples were no longer mixed by changing direction as they passed from one stage to the next and cells no longer passed through vias that effectively act as two-dimensional constrictions. Next we investigated the effect of reducing channel dimensions throughout the device such that minimum widths in the final stages were 100 and 75 µm minimum width. The 100 µm device results were similar to the original multilayer device after both 3 and 10 passes. Decreasing channel width further to 75 µm provided the most cells after both 3 and 10 device passes through the device, more than doubling the number of new single cells generated in comparison to the original multilayer device. While single cell results were generally associated with high variability, we note that a contributing factor was the challenge of reproducibly generating similar initial aggregate populations from experiment to experiment.

To obtain information about aggregate populations, cell suspensions were imaged under phase contrast before and after device treatment (Fig. 2b). Micrographs were then processed to identify contiguous cellular units and determine their area. Results are plotted as a histogram in Fig. 2c. The control and device-treated MCF7 populations were similar up to ~200 pixels^2^, or 750 μm^2^, and from this point the control had significantly more events. After extensive cross-referencing with the original micrographs, we defined four population categories: single cells, clusters (2 to 3 cells), small aggregates (~4 to 10 cells), and large aggregates (>10 cells). These categories were defined primarily based on area values, however we did observe overlap between the single cell and cluster populations, and thus included aspect ratio as a secondary metric. The number of events in each category were summed for each treatment condition, and the results are presented for 10 pass device conditions in Fig. 2d. Single cells constituted less than half of the total events for controls, but increased to as high as ~70% with device treatment. Small and large aggregates both represented approximately 10% of the events in controls. Device treatment decreased small aggregates by approximately half and large aggregates to <1% for device cases. Clusters initially represented 35% of controls, and decreased to around 25% for all device cases. Similar results were observed after 3 passes (see Supplementary Information, Fig. S3), but with slightly higher percentages of clusters and aggregates.

### Optimization of channel geometry

**Figure 3:**
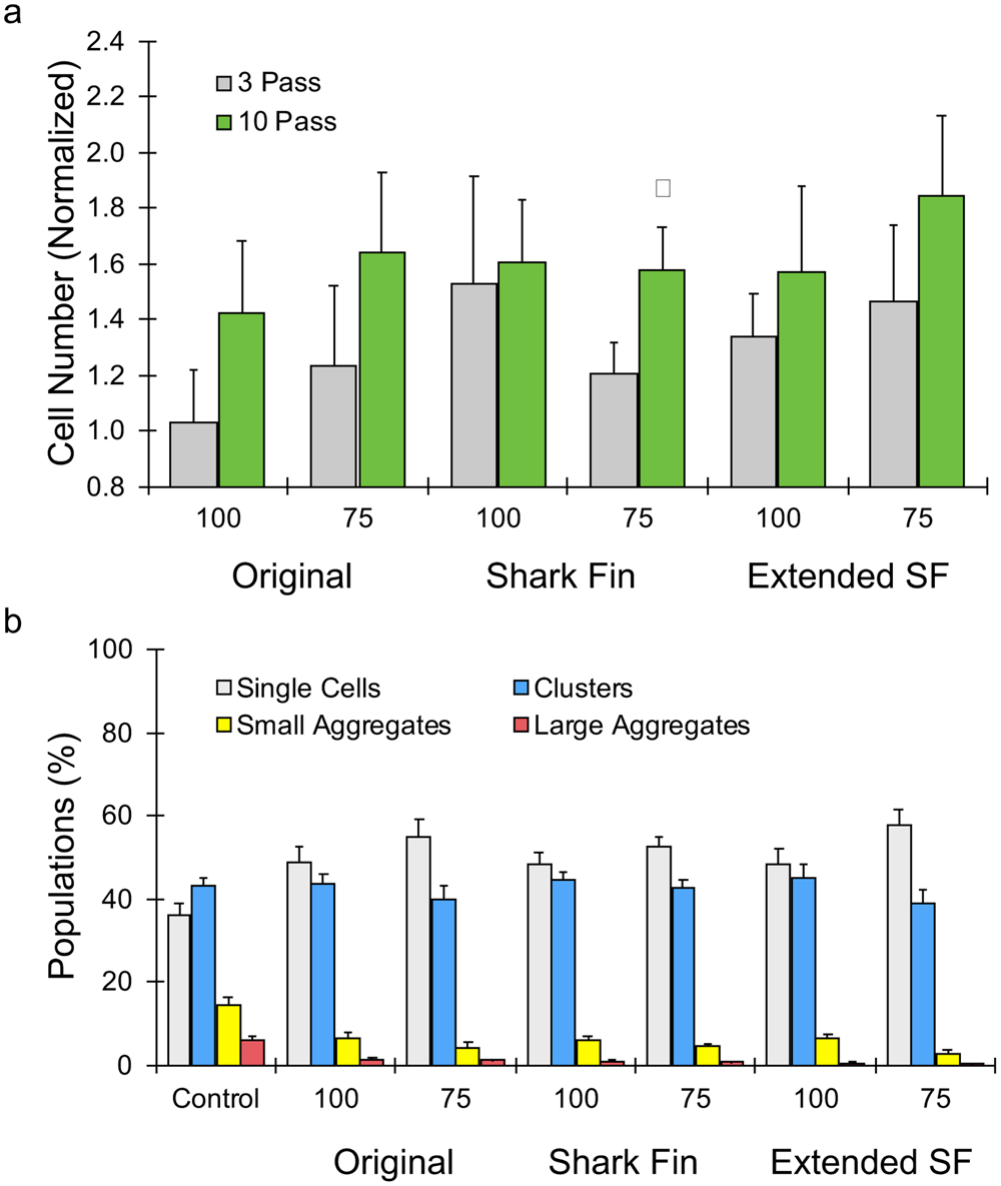
Optimization of channel geometry. (**a**) Normalized MCF7 cell counts obtained for single layer devices with the original, shark fin, and extended shark fin (extended SF) channel geometries with 100 and 75 μm channel minimum widths. All devices produced more single cells after 10 passes, but only the shark fin and extended SF were significant. (**b**) Cell populations plotted as percent total for the control and various SL devices after 10 passes. The extended SF design with 75 µm minimum width displayed the highest single cell and lowest cluster/aggregate percentages. Error bars represent standard errors from at least three independent experiments. *indicates p < 0.05 relative to non-device processed control (value = 1).

Next we examined the influence of channel shape, namely the new shark fin and extended shark fin expansion geometries. Experiments were again performed using the MCF7 cancer aggregate model. The original design was again tested to enable direct comparison, and generally yielded similar results for both channel widths (Fig. 3a). The shark fin design was slightly better than the original at 100 µm minimum width, but there was no difference in performance at 75 µm minimum width. The extended shark fin was most promising, with elevated cell counts across all dimensions and pass numbers, and the highest overall level of single cells produced. While the trends were clear, it should be noted that differences between the device conditions were not statistically significant due to variability between the experiments. As discussed previously, this variability can be attributed in part to difference in initial aggregate populations. We further quantified cell clusters and aggregates in micrographs, and the cell area histograms are shown in the Supplementary Information, Fig. S4. For all device conditions, nearly all large aggregates and most small aggregates were dissociated, while small clusters remained in the range of 40–45%. Single cell percentages were elevated from 35 to ~50% for most cases. The extended shark fin with 75 µm minimum width provided optimal results, with the highest percentage of single cells at 60% and lowest percentages of all aggregate species.

### Validation of device performance using murine kidney tissue

**Figure 4:**
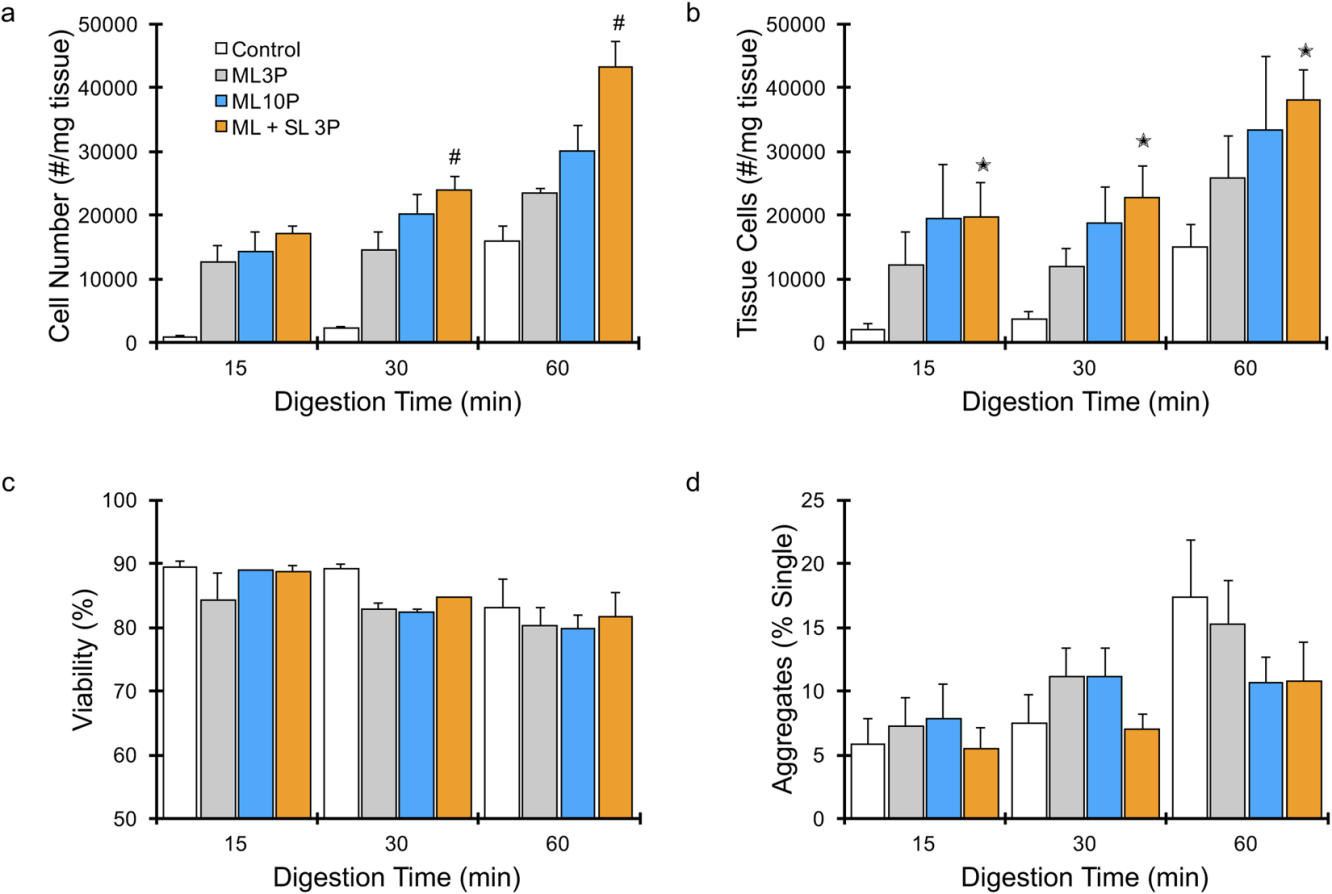
Analysis of dissociated murine kidney cell suspensions. Mouse kidneys were minced, digested with collagenase, and processed under three device conditions: original ML device for 3 and 10 passes and the combination of ML plus SL (extended shark fin, 75 µm) devices for 3 passes. (**a**) Cell numbers were determined using a cell counter after lysing red blood cells, and showed that all device conditions produced significantly more single cells than digestion alone (p < 0.05 for all cases). The ML and SL coupled devices provided the highest cell counts at all digestion times. Flow cytometry was also used to directly analyze cell suspensions so as to identify tissue cells and assess viability. (**b**) The number of single tissue cells recovered matched the cell counter results, but with higher variability. (**c**) Tissue cell viability was not affected by device processing at 15 and 60 min. There was a non-significant trend toward a decrease in cell viability at 30 min, although this is difficult to interpret given the 15 min and 60 min data. (**d**) Relative number of tissue aggregates with respect to single tissue cells increased with digestion time, and was lowest for the dual device condition. Error bars represent standard errors from at least three independent experiments. *Indicates p < 0.05 relative to control at the same digestion time. ^#^Indicates p < 0.05 compared to ML 3 P condition.

As a final dissociation test, we evaluated performance of the original multilayer and new optimized extended shark fin device using tissue obtained from freshly resected murine kidneys. Kidneys are challenging to dissociate because the tissue contains a dense array of blood vessels and tubules, and epithelial cells have tight intercellular junctions and specialized basement membranes^19,36^. Fresh kidneys were harvested from mice, cut with a scalpel into histologically similar sections, weighed, and then further minced into ~1 mm^3^ pieces. Samples were then digested with collagenase for 15, 30, or 60 min in a conical tube under constant agitation, and vortexed every 5 min. Following digestion, samples were processed using three different device conditions: 3 passes through the multilayer device, 10 passes through the multilayer device, or 3 passes through the multilayer and single layer (extended shark fin, 75 µm minimum width) devices that were connected in series. We did not test the single layer device alone due to concerns about potential clogging in the narrower channels, and instead used it only after the multilayer device as a final polishing step. Moreover, we only used 3 passes with the coupled device condition because sample treatment was effectively doubled, and thus we could maximize operational efficiency. Control samples were mechanically treated by vortexing and pipetting after collagenase treatment, per routine procedure. All digested cellular suspensions were treated with DNase, filtered through a 40 μm cell strainer, and separated into two representative portions. One portion was treated with red blood cell lysis buffer and analyzed using the cell counter, while the other portion was reserved for flow cytometric analysis. For controls, cell recovery increased progressively from ~1000 to 2000 cells/mg tissue between 15 and 30 min digestion times, but jumped to ~16,000 cells/mg at 60 min (Fig. 4a). All three device conditions produced dramatically more cells than controls at each digestion time. After 15 min, three passes through the multilayer device yielded 10-fold more cells than the control. Increasing the number of device passes to 10 had little effect, but co-processing with the single layer device yielded 30% more cells. Findings at the 30 and 60 min digestion times followed similar trends, but with progressively smaller differences with respect to the controls because cells were better liberated enzymatically. However, there were also progressively larger differences between the device conditions. In terms of maximum cell yield achieved after 60 min digestion, the multilayer 3 pass, multilayer 10 pass, and dual device 3 pass conditions produced 50%, 100%, and 200% more cells than the control, respectively. Notably, the dual device produced more cells after a brief 15 min digestion than the control after 60 min digestion.

The remaining portion of each sample was analyzed by flow cytometry using a panel of fluorescent stains to distinguish tissue cells from non-cellular debris, red blood cells and leukocytes, as well as assess viability, as we recently described^37^. The number of tissue cells obtained per mg tissue are presented in Fig. 4b, and generally corroborate cell counter results, but with somewhat higher variability. It is unclear whether this variability was due to the greater number of processing steps and time required to label cells with probes, lower reliability of flow cytometry for cell counting, or a change in cell appearance that affected the gating scheme. Tissue cell viability remained similar for all conditions at the 15 and 60 min digestion times, although there was a non-significant trend toward a decrease in cell viability at 30 min that is difficult to interpret given the 15 min and 60 min data (Fig. 4c). The recovery of red blood cells and leukocytes followed the same trends as tissue cells (see Supplementary Information, Fig. S6), suggesting that both longer digestion and increased hydrodynamic dissociation provided greater access to, and likely breakdown of, blood vessels. Interestingly, the relative number of tissue aggregates with respect to single cells increased with digestion time in control cell suspensions (Fig. 4d). These were likely clusters and small aggregates, as samples had been filtered with the 40 µm strainer. Aggregate percentage was lowest after using the single layer extended shark fin device, as they were converted into more single cells. These results conclusively demonstrate that hydrodynamic treatment using our microfluidic dissociation device concept dramatically increases cell recovery. This was observed at both short and long digestion times, with devices consistently liberating more single cells. Moreover, our new optimized shark fin design can produce more single cells by efficiently breaking down small aggregates and clusters.

## Conclusion

Here, we show that a single layer polyimide film can be used to fabricate robust microfluidic devices for dissociating cancer cell aggregates and tissues into single cells. We also used this method as a simple, low-cost, and rapid prototyping method to optimize channel features. While the single layer format is generally less effective than a comparable multilayer design, reducing channel width and changing to the new extended shark fin expansion geometry resulted in the most efficient dissociation and highest single cell recoveries that we have observed to date. We also demonstrated, for the first time, using mouse kidney tissue that our microfluidic dissociation device concept can produce dramatically more single cells than digestion alone. We also show that using two devices can produce the best results for tissues, as it would otherwise be hard to span a large enough range of channel sizes to effectively break down the largest tissue fragments so that the device doesn’t clog, while also being able to convert the smallest cell clusters into single cells. Specifically, the combination of the multilayer device with larger channel dimensions and the optimized single layer device with smaller shark fin channels produced significantly more single cells from kidney tissues. We expect that these results will translate to other tissue types, such as softer and more fragile liver tissue or tougher and denser solid tumors, and testing these samples will be the focus of future work. This final device still requires off-chip mincing and digestion, and therefore a major goal will be to integrate with our microfluidic digestion device^37^. We will also continue to explore new designs for our dissociation device, as well as combining with downstream microfluidic devices and operations to process and analyze the single cell suspensions.

## Materials and Methods

### Device fabrication

Dissociation devices were fabricated using laser micro-machined polyimide films following methods similar to our previous work^28^. Briefly, the device was composed of 4 laser micro-machined layers that were laminated together as follows:

1. The top layer was fabricated from 1.5 mm acrylic sheets (McMaster Carr, Elmhurst, IL) with through holes to access the microfluidic layers.
2. The second layer was fabricated from PET films that had silicone based adhesive transfer tape (3M, St. Paul, MN) laminated to both sides. The second layer contained a reservoir for collecting and passing the dissociated samples to the outlet.
3. The third layer featured the branched microfluidic channel structures, which was fabricated by laser micro-machining polyimide films typically used in the flexible circuit industry.
4. The bottom layer was fabricated from PET films having silicone based adhesive transfer tape (3M, St. Paul, MN) on the top side. This layer sealed the microfluidic channel layer from the bottom.

Hose barbs were glued into the top layer of the finished device to provide tubing connectors. 2D channel features were designed in AutoCAD (Autodesk, San Rafael, CA).

### Fluid dynamics simulations

Flow profiles and shear rates within fluidic channels were simulated using COMSOL Multiphysics software (COMSOL, Burlington, MA). Finite element simulations were conducted by coupling the Navier-Stokes equations with the continuity equation under the assumptions of laminar flow and the no-slip boundary condition. Flow rate was either 1 or 12.5 mL/min. Flow velocity was assessed throughout the expansion and constriction regions for stage 5 of all device designs. Shear rate at the centerline, where cells and aggregates are most likely to be present, was also calculated based on flow gradients in the axial and cross-sectional dimensions.

### Cell culture and tissue models

Human breast cancer cell line MCF7 was obtained from ATCC (Manassas, VA) and cultured in standard tissue culture flasks at 37 **°**C in 5% CO_2_ using recommended media: DMEM media containing 10% FBS, 2 mM L-Glutamine, 1 mM sodium pyruvate, 1× non-essential amino acids, 100 U/mL penicillin, 100 μg/mL streptomycin, and 44 U/L Novolin R insulin (Thermo Fisher, Waltham, MA). Prior to experiments, confluent MCF7 monolayers were digested with trypsin-EDTA for 5 min, which was sufficient to liberate cells but also retain a significant number of cellular aggregates. Cell suspensions were washed by centrifugation into PBS containing 1% BSA (PBS+).

Kidneys were harvested from freshly sacrificed C57B/6 or BALB/c mice (Jackson Laboratory, Bar Harbor, ME) that were deemed waste from a research study approved by the University of California, Irvine, Institutional Animal Care and Use Committee (courtesy of Dr. Angela G. Fleischman). A scalpel was used to prepare ~1 cm long x ~1 mm diameter strips of tissue that each contained histologically similar sections of the cortex and medulla. Each strip of tissue was then weighed to determine mass, minced with a scalpel down to ~1 mm^3^ pieces, and placed within a conical tube with 200 μL of 0.25% collagenase type I (Stemcell Technologies, Vancouver, BC). Samples were digested at 37 °C in an incubator under gentle agitation by a rotating mixer for 15, 30, or 60 min, followed by repeated vortexing and pipetting to mechanically disrupt aggregates. Cell suspensions were then treatment with 100 Units of DNase I (Roche, Indianapolis, IN) for 5 min at 37 °C and washed by centrifugation into PBS + .

### Microfluidic dissociation

Devices were prepared by affixing 3″ PVC 1/32ID tubing (Nalgene, Rochester, NY) to the hose barbs at the inlet and outlet. A 3-way stopcock was used to connect the device to a syringe containing the cell sample and a buffer reservoir containing 1 mL PBS + . Immediately prior to experiments, devices were filled with SuperBlock blocking buffer (Thermo Fisher, Waltham, MA), incubated for 15 minutes, and flushed with PBS+. Cell suspensions were then loaded into the sample syringe and administered to the device using a syringe pump (Harvard Apparatus, Holliston, MA) at a flow rate of 12.5 mL/min. Multiple device passes were achieved by reversing the flow of the syringe pump to replace the sample back into the inlet syringe. This process was repeated for the indicated number of passes. Finally, devices were flushed with 1 mL PBS+ to wash out remaining cells.

### Cell counting and imaging

Cell concentration was determined using a Moxi Z cell counter with type S cassettes (Orflo, Hailey, ID), which utilizes the Coulter principle for cell counting. Cell counts were performed directly on MCF7 suspensions. For kidney samples, one fifth of each suspension was transferred to a new conical tube and incubated with red blood cell lysis buffer containing ammonium chloride, potassium carbonate, and EDTA (BioLegend, San Diego, CA) for 5 min at room temperature prior to obtaining the cell count. MCF7 cell suspensions remaining after the cell count were added to a 12-well plate and imaged using a Hoffman phase contrast microscope with a 4× objective. Raw images were converted to binary using MATLAB (MathWorks, Natick, MA), and all distinct cellular units were identified and outlined using ImageJ. Finally, the area of each cellular unit was calculated and classified into the following categories: single cells (20 to 80 pixels^2^ or 75 to 300 µm^2^), clusters (80 to 200 pixels^2^ or 300 to 750 µm^2^), small aggregates (200 to 300 pixels^2^ or 750 to 1120 µm^2^), and large aggregates (>300 pixels^2^ or >1120 µm^2^). Comparing these categories back to the micrographs, clusters correlated to ~2 to 3 cells, small aggregates contained ~4–10 cells, and large aggregates included >10 cells.

### Flow cytometric analysis

Remaining murine kidney cell suspensions were added to FACS tubes (Corning, Corning, NY) and resuspended in PBS + . Samples were first labeled and analyzed as we recently described^37^. Briefly, cells were co-stained with 0.5× CellMask Green (Thermo Fisher, Waltham, MA) and 2.5 μg/mL anti-mouse CD45-PE monoclonal antibody (clone 30-F11, BioLegend, San Diego, CA) at 37 **°**C for 20 minutes, washed twice with PBS + by centrifugation, and co-stained with 12.5 μM DRAQ5 (BioLegend) and 5 μg/mL 7-AAD (BD Biosciences, San Jose, CA) on ice for at least 15 minutes prior to analysis on an Accuri Flow Cytometer (BD Biosciences). Flow cytometry data was compensated and analyzed using FlowJo software (FlowJo, Ashland, OR). Compensation settings were established using the minced control that was digested for 60 min, CompBeads (3.0–3.4 μm diameter, BD Biosciences), and preparations with and without the above stains or an isotype matched, PE-conjugated monoclonal antibody (Rat IgG2b clone RTK4530, BioLegend) that was used as a control. Gates for each positive and negative subpopulation were defined using FlowJo to automatically calculate the compensation matrix. Finally, a sequential gating scheme was used to identify live and dead tissue cells from red blood cells, leukocytes, and non-cellular debris (see Supplementary Information, Fig. S5).

### Statistics

Data are represented as the mean ± standard error determined from at least three independent experiments. P-values were calculated based on mean and standard error values using students t-test.

### Data Availability

All data generated or analysed during this study are included in this published article (and its Supplementary Information files).

## Electronic supplementary material

Supplementary Information
