# Supplementary material for: Microfluidic channel optimization to improve hydrodynamic dissociation of cell aggregates and tissue
Source: Sci Rep. 2018 Feb 9;8:2774. doi: 10.1038/s41598-018-20931-y (PMC5807353; doi:10.1038/s41598-018-20931-y)
Supplement: Supplementary file 1 — Supplementary Information [file 41598_2018_20931_MOESM1_ESM.pdf]

Supplementary Information

Microfluidic channel optimization to improve hydrodynamic dissociation of cell aggregates and tissue

Xiaolong Qiu,[a] Jen-Huang Huang,[f] Trisha M. Westerhof,[c] Jeremy A. Lombardo,[a] Katrina M. Henrikson,[a] Marissa Pennell,[a] Pedram P. Pourfard,[a] Edward L. Nelson,[cde] Pulak Nath,[g] and Jered B. Haun[abe]*

[a.] Department of Biomedical Engineering, Henry Samueli School of Engineering, University of California Irvine, Irvine, CA 9269, USA

[b.] Department of Chemical Engineering and Materials Science, Henry Samueli School of Engineering, University of California Irvine, Irvine, CA 92697, USA

[c.] Department of Medicine, Division of Hematology/Oncology, School of Medicine, University of California, Irvine, Irvine, CA 92697, USA

[d.] Department of Molecular Biology and Biochemistry, Ayala School of Biological Sciences, University of California, Irvine, Irvine, CA 92697, USA.

[e.] Chao Family Comprehensive Cancer Center, University of California Irvine, Irvine, CA 92697, USA

[f.] Department of Chemical Engineering, National Tsing Hua University, Hsinchu, Taiwan

[g.] Applied Modern Physics, Los Alamos National Laboratory, Los Alamos, NM 87545, USA

Table of Contents

Figure S1 Channel resolution of multilayer and single layer devices.

Figure S2 Channel specifications for each design

Figure S3 Cell populations for multilayer and single layer devices after 3 passes.

Figure S4 Cell populations after 3 passes for different channel geometries.

Figure S5 Flow cytometry gating scheme.

Figure S6 Red blood cell and leukocyte populations in murine kidney cell suspensions.

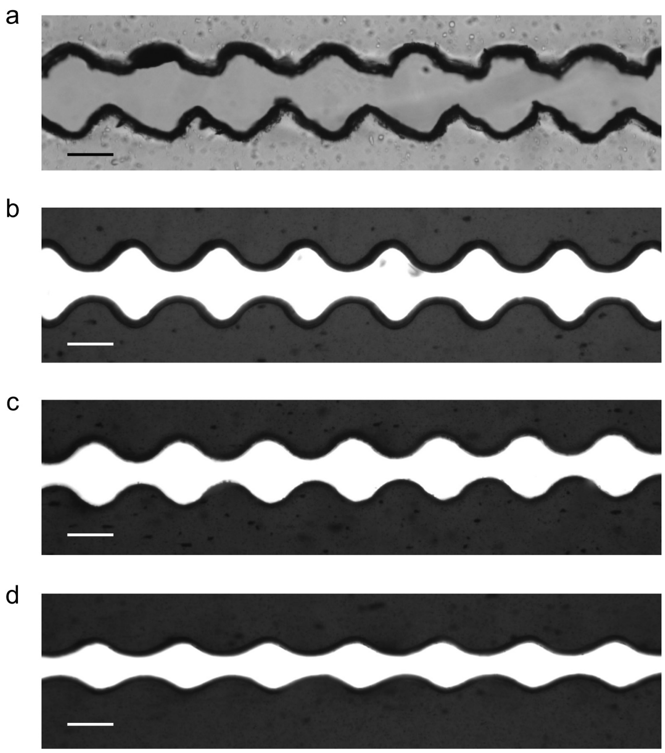

Figure S1. Channel resolution of multilayer and single layer devices. Micrographs were taken of the fifth and final stage of the (a) multilayer device and single layer devices at (b) 125, (c) 100, and (d) 75 μm minimum channel width. The multilayer device was designed to have 125 μm minimum width. Single layer, polyimide films produced far superior feature resolution. Scale bars indicate 200 μm.

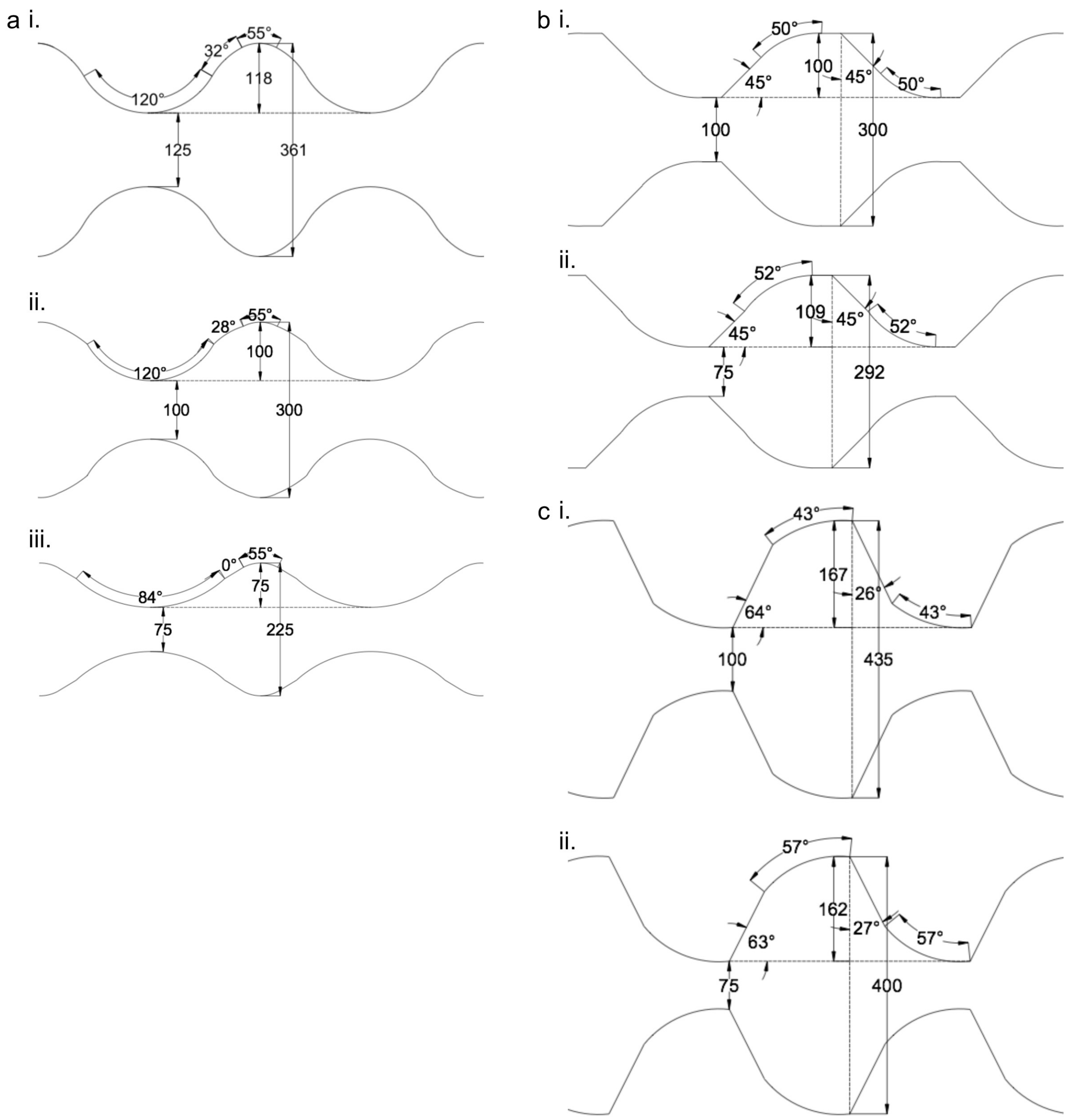

Figure S2. Channel specifications for each design. (a) Original design at (i) 125, (ii) 100, and (iii) 75 µm minimum width in stage 5. (b) Shark fin design at (i) 100 and (ii) 75 µm minimum width in stage 5. (c) Extended shark fin (ESF) design at (i) 100 and (ii) 75 µm minimum width in stage 5.

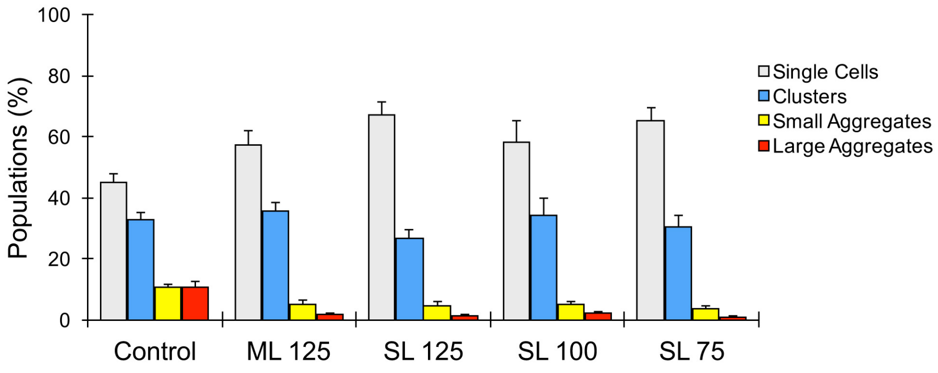

Figure S3. Cell populations for multilayer and single layer devices after 3 passes. Cell populations were determined from micrographs and plotted as percent total for the control, ML device, and various SL devices after 3 passes. Results were similar to those after 10 passes presented in Fig. 2d, but with slightly larger cluster and aggregate populations. Error bars represent standard errors from at least three independent experiments.

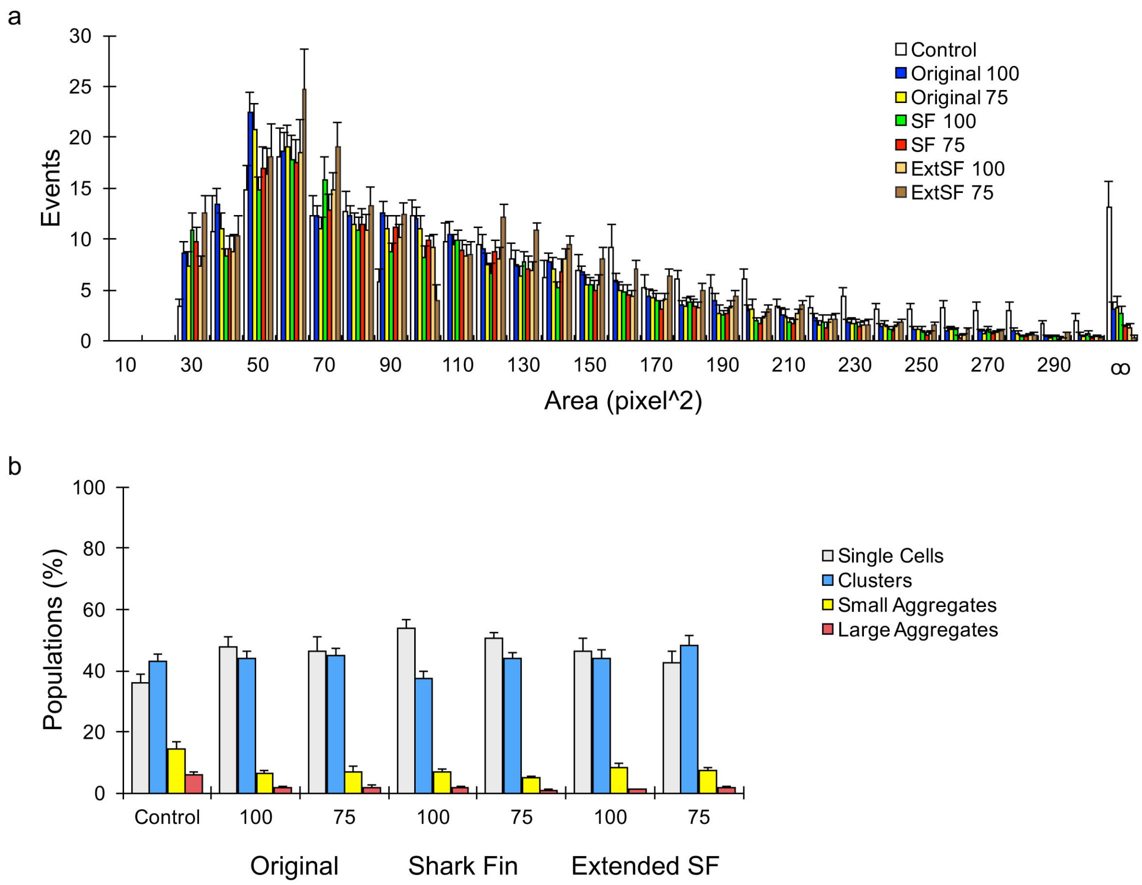

Figure S4. Cell populations after 3 passes for different channel geometries. (a) Cell unit area histogram used to determine cell populations in MCF7 suspensions based on image analysis of micrographs. Data was combined between 3 and 10 pass conditions. (b) Cell populations plotted as percent total for the control and various SL devices after 3 passes. Results were similar to those after 10 passes presented in Fig. 3b, but with slightly larger cluster and aggregate populations. Error bars represent standard errors from at least three independent experiments.

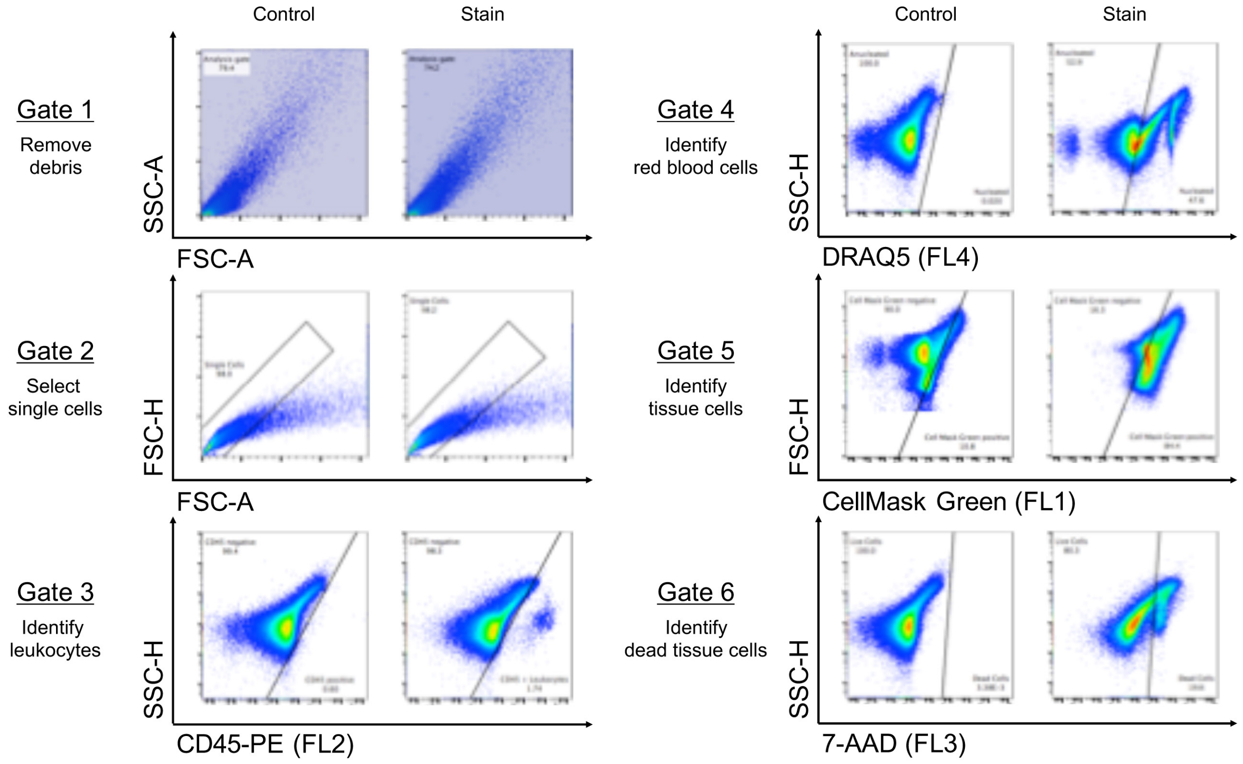

Figure S5. Flow cytometry gating scheme. Cell suspensions obtained from digested murine kidney samples were stained with a four-probe panel and analyzed using flow cytometry. Controls were treated only with an isotype matched (IgG2b), PE-conjugated antibody. Acquired data was assessed using a sequential gating scheme. First, an FSC-A vs. SSC-A gate (Gate 1) was used to exclude debris near the origin. Gate 2 was based on FSC-A vs. FSC-H, and was used to select single cells. Gate 3 distinguished CD45+ leukocytes based on CD45-PE signal in FL2-A vs. SSC-H plots. The CD45 cell subset was further divided into anucleate RBCs and nucleated tissue cell subsets based on signal from the DRAQ5 nuclear stain in FL4-A vs. SSC-H plots. The cellularity of nucleated tissue cells of interest was validated based on signal of the cell membrane dye CellMask Green in FL1-A vs. FSC-H plots. Finally, live and dead tissue cells were discriminated based on 7-AAD signal in FL3-A vs. SSC-H plots. All gates were established using the minced control that was digested for 60 min. Heat treated cells were used as a positive control to confirm appropriate 7-AAD signal for dead cells.

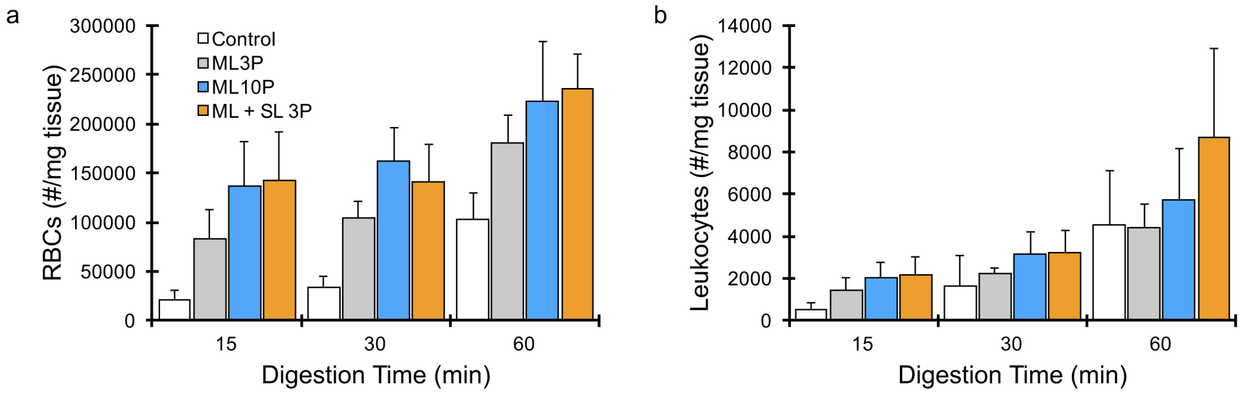

Figure S6. Red blood cell and leukocyte populations in murine kidney cell suspensions. Flow cytometry was used to identify (a) red blood cells based on the lack of the nuclear stain DRAQ5 and (b) leukocytes based on positive CD45 stain. The recovery of both cell types increased with digestion time and device processing, in a similar manner as single tissue cells. Error bars represent standard errors from at least three independent experiments.
